# Septic Shock Induced by Bacterial Prostatitis with* Morganella morganii* subsp.* morganii* in a Posttransplantation Patient

**DOI:** 10.1155/2015/850532

**Published:** 2015-12-21

**Authors:** Xiaofan Li, Jianhui Chen

**Affiliations:** ^1^Department of Hematology, Fujian Institute of Hematology, Fujian Provincial Key Laboratory on Hematology, Fujian Medical University Union Hospital, Fuzhou 350001, China; ^2^Department of Urology, Fujian Medical University Union Hospital, Fuzhou 350001, China

## Abstract

Bacterial infection is a common complication after Hematopoietic Stem Cell Transplantation (HSCT).* Morganella morganii* is ubiquitous Gram-negative facultative anaerobe, which may cause many kinds of opportunistic infection. Herein we report a case of a 55-year-old man who presented with frequent urination, urgency, and mild pain that comes and goes low in the abdomen and around the anus. The patient had a medical history of chronic prostatitis for 4 years. He received HLA-matched sibling allo-HSCT because of angioimmunoblastic T-cell lymphoma 29 months ago. The routine examination of prostatic fluid showed increased leukocytes and the culture of prostatic fluid showed* Morganella morganii* subsp.* morganii*. The patient developed chills and fever 18 hours after examination. Both urine culture and blood culture showed* Morganella morganii* subsp.* morganii*. The patient was successfully treated with antibiotic therapy and septic shock management. Taken together,* Morganella morganii* should be considered a possible pathogen when immunocompromised patients develop prostatitis. Also, prostatic massage could be a possible trigger of septic shock induced by* Morganella morganii* subsp.* morganii* in a posttransplantation patient.

## 1. Introduction

Hematopoietic stem cell transplant (HSCT) is a curative treatment to hematologic malignancies [1]. Posttransplant patients are in immunocompromised status and bacterial infection is common after hematopoietic stem cell transplant [2].* Morganella morganii* is ubiquitous Gram-negative facultative anaerobe. It may cause many kinds of opportunistic infection, including prostatitis [3]. Several mechanisms of development of bacterial prostatitis are either known or postulated to occur. Prostatic inoculation with large bowel organisms, ascending infection, and haematogenous and lymphatogenous spread as well as direct spread from pelvic foci of infection may contribute to the routes of prostatic involvement [4].

Herein we report a case of septic shock induced by bacterial prostatitis in a posttransplantation patient with immunosuppression status, in which prostate plays a sanctuary role as the primary infection foci for* Morganella morganii*.

## 2. Case Report

A 54-year-old Chinese Han male had frequent urination and urgency for 4 months accompanied with mild pain that comes and goes low in the abdomen and around the anus. This chronic pelvic pain syndrome was mild and did not affect his normal life except that he had nocturia 2-3 episodes/night. He visited the outpatient department with a worsening syndrome of frequent urination and urgency for 5 days. He had no fever and no chill and had nocturia 4-5 episodes/night. He denied recent genitourinary instrumentation or new sex partner. Four years ago, he was diagnosed with chronic prostatitis, which was cured by 4 weeks of antibiotic and nonsteroidal anti-inflammatory drugs (NSAIDs).

It is noteworthy that he had a medical history of angioimmunoblastic T-cell lymphoma (AITL) and received HLA-matched sibling allo-HSCT 29 months ago. During HSCT, the patient was stable without classical symptoms of prostatitis, showing no frequent urination, urgency, or difficulty in urinating. He recovered well from HSCT and took methylprednisolone 6 mg every day to treat chronic graft versus host disease.

Given the reoccurring symptom of prostatitis and negative result of routine urine test, prostatic massage was performed to obtain prostatic fluid for test and culture. Digital rectal examination shows normal except for light tenderness. The routine examination of prostatic fluid showed increased leukocytes (30 white blood cells/high power field) and decreased lecithin body (about 50%). By suspicion of bacterial prostatitis, cefixime was used to treat the patient. Later that day, 18 hours after prostatic massage and examination, he developed urgency, urinary frequency (every 1-2 hours), and nocturia (7-8 episodes/night) accompanied by fever, chills, rigors, and headache. In the emergency room, he had a fever of 39.3°C. His mucous membranes were pale. His pulse rate was 113/minute and BP 80/43 mmHg. Examination of the abdominal and genitalia was normal. Blood routine test showed the following: WBC: 15.06 × 10^9^/L, N%: 67, Hb: 138 g/L, and Plt 232 × 10^9^. Urine test demonstrated positive result of bacteria, white blood cells, and protein. X-ray of chest showed normal. Ultrasound showed mild enlargement of the prostate with prostate calcification. Midstream specimen of urine and blood samples for culture and sensitivity were obtained before antibiotic. Given the patients immunosuppression status, imipenem (500 mg q6h) was used as an initial empirical treatment. Septic shock management was also applied immediately after admission. The culture of prostatic fluid showed >10^5^ colony forming units/mL of* Morganella morganii*. Both urine culture and blood culture also demonstrated* Morganella morganii* subsp.* morganii*, which was sensitive to imipenem and levofloxacin. After the above treatment, the patient showed absence of fever and no detectable* Morganella morganii* colony in both urine culture and blood. After two weeks of imipenem followed by 4 weeks of levofloxacin, the patient recovered from septic shock and still remained well without any pain or any urinary symptoms.

## 3. Discussion

*Morganella morganii* is an opportunistic pathogen, which may cause many kinds of opportunistic infection, such as urinary tract infection and wound infection [3]. It is also associated with other diseases, such as arthritis [5]. Lin et al. [6] reported that, in* Morganella morganii* bacteremia, the urinary tract was the major portal of entry, followed by the hepatobiliary tract, skin, and soft tissue. In the present case, the septic shock was induced by* Morganella morganii* prostatitis.

Patients after HSCT are usually in immunocompromised status and are prone to bacterial infection. Opportunistic infection in post-HSCT patients can be symptomless, more like a chronic disease. In the present case, the patient had a medical history of chronic prostatitis. According to the reoccurring urinary symptoms for 4 months, premassage urine test normal, and the culture of prostatic fluid positive, he was diagnosed with chronic prostatitis (category II) [7].

Aerobic Gram-negative bacilli are the predominant pathogens in bacterial prostatitis.* E. coli* cause 50%–80% of cases; other pathogens include Enterobacteriaceae (e.g.,* Klebsiella* and* Proteus*),* Enterococcus* species, and nonfermenting Gram-negative bacilli (e.g.,* Pseudomonas* species) [8]. In this case,* Morganella morganii* is Gram-negative bacilli belonging to the family Enterobacteriaceae. The main reason for this opportunistic infection is the immunosuppression status after HSCT. Also, the patient had a medical history of chronic prostatitis. It may damage the natural defenses mechanism against infection of the prostate gland. The ultrasound showed prostate calcification, indicating that the possible poor drainage of secretions may contribute to the inflammation.

The approach to treating bacterial infection of the prostate largely centers on appropriately selected sensitive antibiotic therapy. Treatment of chronic bacterial prostatitis has been evaluated in clinical trials mainly with fluoroquinolones. Alternative antimicrobial agents were cotrimoxazole, beta lactams, and tetracyclines [9].* Morganella morganii* is usually susceptible to quinolones such as ciprofloxacin, aminoglycosides, such as gentamicin, amikacin, tobramycin, chloramphenicol, cotrimoxazole, aztreonam, and other carbapenems [10].* Morganella morganii* are resistant to penicillins and many cephalosporins through the formation of ESBL and Amp C-beta lactamase production [11]. In the present case, the patient recovered soon after sensitive antibiotic treatment.

Given the immunocompromised status, the prostatic massage could be a possible trigger of septic shock. Prostatic massage is a routine exam for the diagnose of chronic prostatitis (category II). In the present case, septic shock was associated with prostatic massage. We should have noticed the specialty of post-HSCT immunocompromised status and prescribed broad-spectrum antibiotic specific to Gram-negative bacteria, which might avoid the bloodstream infection.

In conclusion, we report a case of septic shock induced by bacterial prostatitis with* Morganella morganii* subsp.* morganii* in a posttransplantation patient. We report this case to create the awareness among clinicians that* Morganella morganii*, even though uncommon, can be a cause of chronic prostatitis. In immunocompromised patients, as it may have a slow insidious onset, with minimal characteristic signs and symptoms, a high index of suspicion is required. Also, prostatic massage to those patients should be cautious.
